# Engaging a national-scale cohort of smart thermometer users in participatory surveillance

**DOI:** 10.1038/s41746-023-00917-5

**Published:** 2023-09-20

**Authors:** Yi-Ju Tseng, Karen L. Olson, Danielle Bloch, Kenneth D. Mandl

**Affiliations:** 1 Computational Health Informatics Program, Boston Children’s Hospital, Boston, MA USA; 2 Department of Computer Science, National Yang Ming Chiao Tung University, Hsinchu, Taiwan; 3 Department of Pediatrics, Harvard Medical School, Boston, MA USA; 4 Kinsa Inc, San Francisco, CA USA; 5 Department of Biomedical Informatics, Harvard Medical School, Boston, MA USA

**Keywords:** Viral infection, Population screening, Respiratory signs and symptoms

## Abstract

Participatory surveillance systems crowdsource individual reports to rapidly assess population health phenomena. The value of these systems increases when more people join and persistently contribute. We examine the level of and factors associated with engagement in participatory surveillance among a retrospective, national-scale cohort of individuals using smartphone-connected thermometers with a companion app that allows them to report demographic and symptom information. Between January 1, 2020 and October 29, 2022, 1,325,845 participants took 20,617,435 temperature readings, yielding 3,529,377 episodes of consecutive readings. There were 1,735,805 (49.2%) episodes with self-reported symptoms (including reports of no symptoms). Compared to before the pandemic, participants were more likely to report their symptoms during pandemic waves, especially after the winter wave began (September 13, 2020) (OR across pandemic periods range from 3.0 to 4.0). Further, symptoms were more likely to be reported during febrile episodes (OR = 2.6, 95% CI = 2.6–2.6), and for new participants, during their first episode (OR = 2.4, 95% CI = 2.4–2.5). Compared with participants aged 50–65 years old, participants over 65 years were less likely to report their symptoms (OR = 0.3, 95% CI = 0.3–0.3). Participants in a household with both adults and children (OR = 1.6 [1.6–1.7]) were more likely to report symptoms. We find that the use of smart thermometers with companion apps facilitates the collection of data on a large, national scale, and provides real time insight into transmissible disease phenomena. Nearly half of individuals using these devices are willing to report their symptoms after taking their temperature, although participation varies among individuals and over pandemic stages.

## Introduction

Participatory surveillance systems crowdsource individual reports to rapidly assess population health phenomena, potentially yielding a timely signal that complements traditional population health surveillance^1–3^. These systems have proven useful for infectious diseases, including influenza-like illness (ILI)^4^, vector-borne diseases^5^, foodborne illnesses^6^, and COVID-19^7–9^.

Common platforms underlying participatory surveillance systems include social media sites, the Web, smartphone apps, and connected devices^2,10^. One of the most crucial challenges is recruitment and retention of a large cohort of participants reflecting the population of interest^3,11,12^. The value of these systems increases dramatically when more participants join and persistently contribute^4,9^. Willingness to participate may be affected by marketing and recruitment efforts^11^, individual demographic characteristics^13–15^, population disease levels^15^, and media coverage^16^. Furthermore, the behavior of checking symptoms or taking body temperature may be affected by one’s level of anxiety regarding infectious diseases^17,18^.

We sought to analyze the level of and factors associated with engagement in participatory surveillance among a national-scale cohort of individuals using smartphone-connected digital thermometers. When recording temperatures, participants can use the companion smartphone app to report symptoms and assign readings to profiles with self-reported demographic information. In turn, users are provided with basic health guidance developed by clinicians. Temperature readings from commercially available smart thermometers are known to be effective in forecasting influenza^19^ and ILI^20^, as well as discerning within-household infection transmission dynamics^21^.

## Results

### Characteristics of the participants

**Fig. 1:**
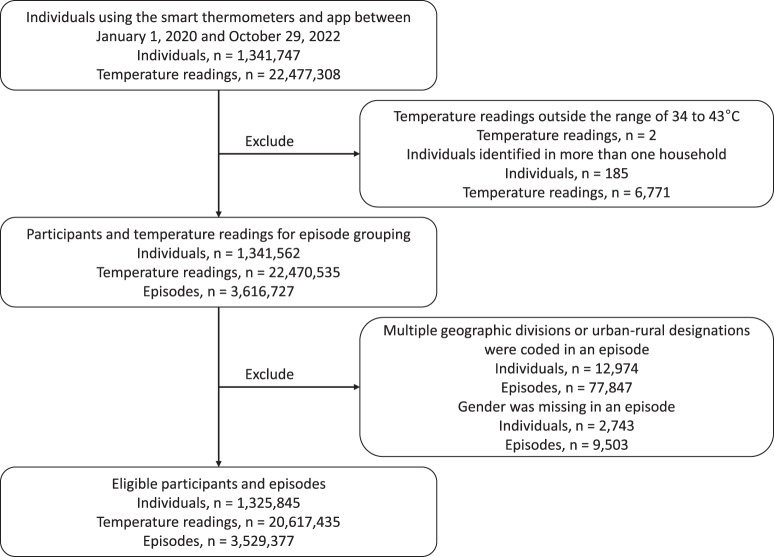
Flow diagram of participants and temperature readings. Of 1,341,747 individuals using the smart thermometers and app between January 1, 2020 and October 29, 2022, 1,325,845 individuals met the inclusion criteria. These eligible participants took 20,617,435 temperature readings, yielding 3,529,377 episodes of consecutive temperature readings during the study period.

In total, 1,325,845 participants took 20,617,435 temperature readings during the study period (January 1, 2020 to October 29, 2022), yielding 3,529,377 episodes of consecutive temperature readings (Fig. 1).

Demographics for distinct participants are shown in Table 1. Demographic and other characteristics of episodes are shown in Tables 1, 2. Most participants (758,498, 57.2%) contributed one temperature reading episode during the study period. There were 218,614 (16.5%) participants with 2, and 348,733 (26.3%) with 3 or more. The median interval between the start of the first and last episodes of participants with 2 episodes was 42 days (IQR [15,122]), and those with or 3 more was 231 (IQR = [99, 455]) days. For almost half (1,735,805, 49.2%) of the episodes, participants self-reported symptoms (including reports of no symptoms). Children under 12 years old were 35.2% of all participants. The number of episodes per person differed by age group. In post-hoc analyses, the median number of episodes per person for children under 12 was higher (1, IQR = [1,3]) than age groups 12–18, 18–35, and 35–50 (1, [1, 2], *p* < 0.001). However, it was lower than groups 50–65 (1, [1, 3]) and over 65 (1, [1, 4]) (*p* < 0.001).

**Table 1:** Participant and Episode Characteristics.

	Participants	Episodes		
Total No.	1,325,845	3,529,377		
		With reported symptoms	Without reported symptoms	*p*-value
No. (%)		1,735,805 (49.2)	1,793,572 (50.8)	
Age, median [IQR]	26.0 [8.0, 42.0]	29.0 [9.0, 47.0]	31.0 [8.0, 54.0]	< 0.001
Age group, No. (%)				< 0.001
Age < = 12	466,471 (35.2)	578,366 (33.3)	584,087 (32.6)	
12 < Age < = 18	96,970 (7.3)	99,430 (5.7)	84,433 (15.9)	
18 < Age < = 35	306,762 (23.1)	352,661 (20.3)	355,597 (12.2)	
35 < Age < = 50	233,932 (17.6)	334,864 (19.3)	265,992 (14.8)	
50 < Age < = 65	130,779 (9.9)	243,977 (14.1)	218,664 (19.8)	
Age > 65	90,931 (6.9)	126,507 (7.3)	284,799 (4.7)	
Gender, No. (%)				< 0.001
Female	721,136 (54.4)	979,045 (56.4)	981,214 (54.7)	
Male	542,899 (40.9)	699,601 (40.3)	726,500 (40.5)	
Other	11,168 (0.8)	9,931 (0.6)	17,699 (1.0)	
Unknown	50,642 (3.8)	47,228 (2.7)	68,159 (3.8)	
US geographic division, No. (%)				< 0.001
East North Central	167,838 (12.7)	221,272 (12.7)	237,136 (13.2)	
East South Central	53,382 (4.4)	68,009 (3.9)	73,008 (4.1)	
Middle Atlantic	195,853 (14.8)	262,848 (15.1)	262,817 (14.7)	
Mountain	84,587 (6.4)	113,256 (6.5)	118,127 (6.6)	
New England	92,265 (7.0)	127,898 (7.4)	129,475 (7.2)	
Pacific	289,331 (21.8)	380,675 (21.9)	379,433 (21.2)	
South Atlantic	203,379 (15.3)	270,384 (15.6)	293,350 (16.4)	
West North Central	81,923 (6.2)	107,566 (6.2)	110,831 (6.2)	
West South Central	152,287 (11.5)	183,897 (10.6)	189,395 (10.6)	
Urban/rural designation, No. (%)				< 0.001
Large central metro	389,547 (32.2)	553,132 (33.9)	557,132 (33.5)	
Large fringe metro	307,975 (25.5)	425,686 (26.1)	449,839 (27.0)	
Medium metro	275,533 (22.8)	357,123 (21.9)	366,183 (22.0)	
Small metro	102,789 (8.5)	132,404 (8.1)	133,712 (8.0)	
Micropolitan	86,276 (7.1)	108,792 (6.7)	102,803 (6.2)	
Noncore	46,423 (3.8)	55,424 (3.4)	54,110 (3.3)	
Household composition, No. (%)				<0.001
Adult only	540,040 (40.7)	758,882 (43.7)	937,295 (52.3)	
Adult and child	462,801 (34.9)	638,153 (36.8)	435,655 (24.3)	
Child only	323,004 (24.4)	338,770 (19.5)	420,622 (23.5)	

Differences in medians were assessed with the Kruskal-Wallis test. Chi-square test was used for categorical variables. Households with children-only profiles were presumed to have adults in the household who were not using the thermometer.

**Table 2:** Episode Characteristics.

	Episodes		
	With reported symptoms	Without reported symptoms	*p*-value
No.	1,735,805	1,793,572	
New participant, No. (%)	767,774 (44.2)	558,071 (31.1)	< 0.001
Fever, No. (%)	425,130 (24.5)	287,414 (16.0)	< 0.001
Severe fever, app skipped symptom page, No. (%)	154,406 (8.9)	133,056 (7.4)	< 0.001
Season			< 0.001
Spring	439,418 (25.3)	571,638 (31.9)	
Summer	361,504 (20.8)	443,911 (24.8)	
Autumn	441,102 (25.4)	374,107 (20.9)	
Winter	493,781 (28.4)	403,916 (22.5)	
Pandemic period, No. (%)			< 0.001
Before pandemic (01/01/2020–02/29/2020)	61,087 (3.5)	83,664 (4.7)	
First outbreak (03/01/2020–05/15/2020)	145,361 (8.4)	270,144 (15.1)	
Second wave (05/16/2020–09/12/2020)	190,840 (11.0)	314,955 (17.6)	
Winter wave (09/13/2020–03/06/2021)	445,190 (25.6)	332,553 (18.5)	
Fourth wave (03/07/2021–07/14/2021)	174,969 (10.1)	158,103 (8.8)	
Delta wave (07/15/2021–12/18/2021)	245,098 (14.1)	199,176 (11.1)	
Omicron BA.1/2 wave (12/19/2021–06/19/2022)	312,114 (18.0)	282,658 (15.8)	
Omicron BA.4/5 wave (06/20/2022–10/29/2022)	161,146 (9.3)	152,319 (8.5)	

Chi-square test was used for categorical variables. If a participant of any age had a high fever (at least 39.4 °C), or a child aged 0 to 3 months had any fever, the symptom report screen within the app was skipped and the user was immediately transferred to a guidance page to address potentially serious medical conditions.

### Thermometer usage and COVID-19 pandemic

**Fig. 2:**
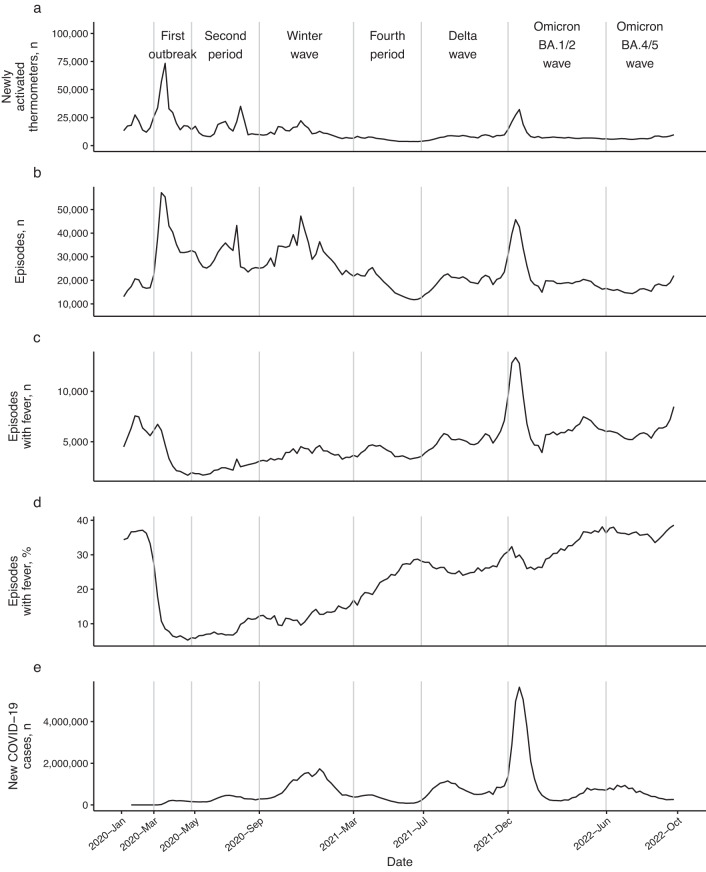
Time series for participatory and viral surveillance sources. Vertical lines are placed at the beginning of each pandemic period. Panels (**a**–**d**) show, from the cohort, newly active thermometers, total episodes, febrile episodes, and the percentage of febrile episodes each week. Panel (**e**) shows the numbers of US COVID-19 cases (Johns Hopkins University Center for Systems Science and Engineering)^52^.

Counts of both newly activated thermometers and temperature reading episodes rose at the beginning of the first outbreak and during the omicron BA.1/2 wave, while the percentage of febrile episodes dropped at the beginning of the first outbreak and steadily increased during the pandemic (Fig. 2).

### Symptom reports and COVID-19 pandemic

**Fig. 3:**
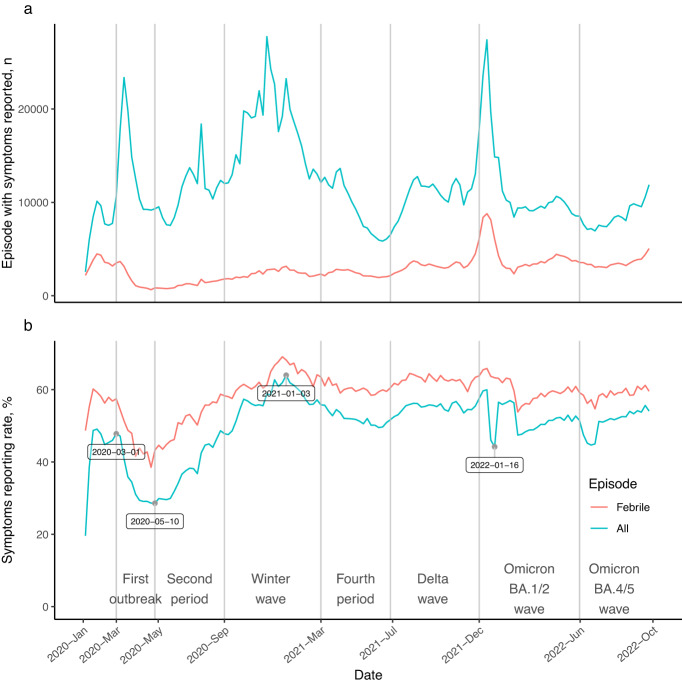
Episodes with symptoms reported and symptom reporting rate. Vertical lines are placed at the beginning of each pandemic period. Panel (**a**) shows the number of episodes with symptoms reported among febrile or all episodes. Panel (**b**) shows the symptom reporting rate among febrile or all episodes. The red lines are the episodes with symptoms reported and symptom reporting rate among febrile episodes, and the green lines are the episodes with symptoms reported and symptom reporting rate among all episodes. The points with dates are the relative high and low reporting rates during the pandemic.

Symptom reporting rates decreased right after the first outbreak (47.8%, 10,695 of 22,356 episodes, week starting from March 1, 2020) until the week starting May 10, 2020 (28.6%, 9316 of 32,613 episodes), and then began steadily increasing. In the winter wave, the reporting rate increased to 64.0% (23,256 of 36,341 episodes) in the week starting from January 3, 2021, then remained around 55% afterward. In the omicron BA.1/2 wave, the symptom reporting rate dropped to 44.2% (14,864 of 33,649 episodes) in the week starting from January 16, 2022, but increased to 56% within a week (Fig. 3). Compared with the weekly symptom reporting rates among all episodes (52.0% (IQR = [47.4–55.6%])), the weekly reporting rates among febrile episodes were considerably higher (59.7% (IQR = [57.1–62.3%])) and relatively stable, with a smaller IQR, during the study period. The decline in reporting rates during the Omicron BA.1/2 wave was not seen among participants with fever (Fig. 3). Among episodes reported with symptoms, 36%, 26%, 16%, and 22% reported 1, 2, 3, and more than 3 symptoms, respectively. The highest rate of multiple coexisting symptoms in an episode was 71% during the Omicron BA.1/2 and BA.4/5 waves. The lowest was 51% during the second period (May 16 to September 12, 2020).

### Factors associated with symptom reports

**Fig. 4:**
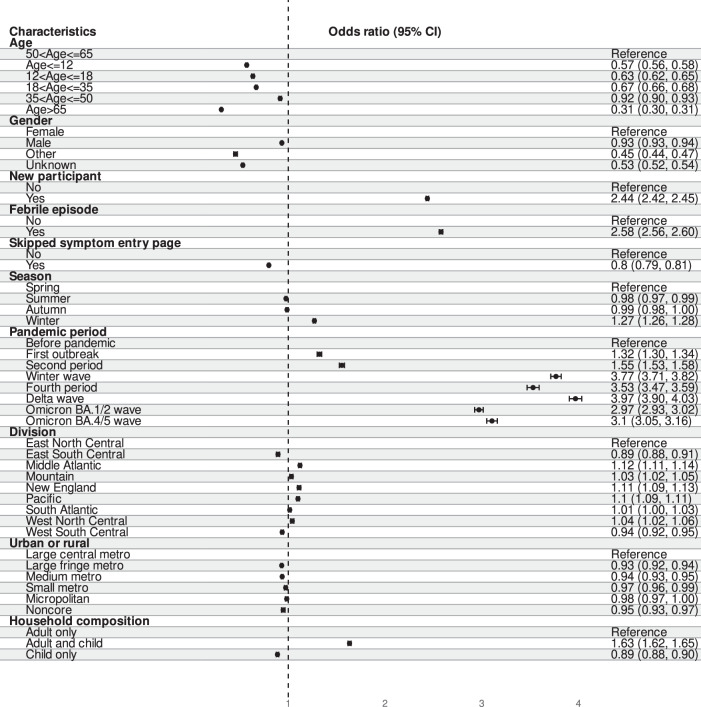
The association between patient characteristics and willingness of individuals to report their symptoms via the smartphone app. Fever was identified by temperature reading, skip symptom entry page came from the app logs, geographic information was based on GPS information or an IP address, and age and gender were self-reported.

We characterized profiles that were either more or less likely to report or record symptom occurrence (Fig. 4). Odds ratios (ORs) were adjusted for all independent variables and covariates. Participants 50 to 65 years old were more likely to report their symptoms, and compared with these participants, participants over > 65 years old were less likely to report their symptoms (OR = 0.3, 95% CI = 0.3–0.3). Symptoms were more likely reported during febrile episodes (OR = 2.6, 95% CI = 2.6–2.6), and for new participants, during their first episode (OR = 2.4, 95% CI = 2.4–2.5). Symptoms were more likely to be reported in winter (OR = 1.3, 95% CI = 1.3–1.3) than in spring. Compared with before the pandemic, participants were more likely to report their symptoms during the pandemic periods, especially the winter wave (September 13, 2020 to March 6, 2021, OR = 3.8, 95% CI = 3.7–3.8), fourth period (March 7 to July 14, 2021, OR = 3.5, 95% CI = 3.5–3.6), delta wave (July 15 to December 18, 2021, OR = 4.0, 95% CI = 3.9–4.0), omicron BA.1/2 wave (December 19, 2021 to June 19, 2022, OR = 3.0, 95% CI = 2.9–3.0) and omicron BA.4/5 wave (June 20 to October 29, 2022, OR = 3.1, 95% CI = 3.1–3.2). Using the east north central division as the reference, participants living in east south central (OR = 0.9, 95% CI = 0.9–0.9) and west south central (OR = 0.9, 95% CI = 0.9–1.0) were less likely to report their symptoms, and participants living in the other divisions were more likely, except south Atlantic. Compared with adult-only households, participants living in a household with both adults and children were more likely to report symptoms (OR = 1.6, 95% CI = 1.6–1.7).

### Symptom trends in the COVID-19 pandemic

**Fig. 5:**
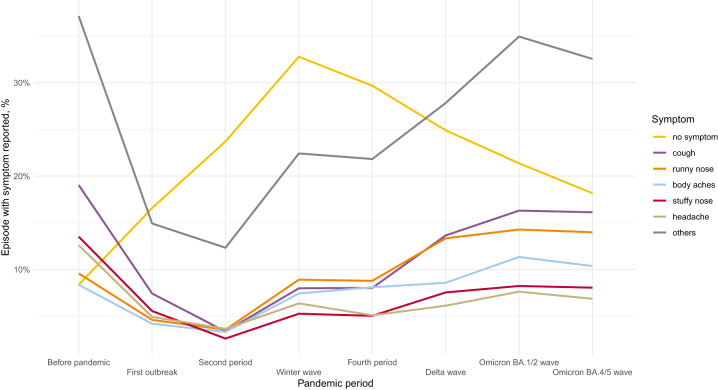
Self-reported symptoms across pandemic periods. The colored lines show the percentage of a specific symptom reported by participants in all temperature reading episode.

There were 3,125,957 symptom reports collected among 1,735,805 episodes (49.2% of all episodes). Of these, 849,486 (48.9%) episodes were associated with reports of no symptoms. The proportion of reports of no symptoms increased during the COVID-19 pandemic periods until the winter wave. In the pandemic periods, the percentage of episodes with reporting no symptoms (24.7% of all episodes, March 1, 2020 to October 29, 2022) was higher, compared with before the pandemic (8.4% of all episodes, January 1 to February 28, 2020, *p* < 0.001). The top five symptoms reported during the COVID-19 pandemic periods from the beginning to the omicron BA.4/5 wave were cough (344,834, 20.6% of episodes with symptom reports), runny nose (323,599, 19.3%), body aches (256,916, 15.3%), stuffy nose (201,588, 12.0%), and headache (199,415, 11.9%). The symptom report trends are shown in Fig. 5.

## Discussion

In a national-scale participatory surveillance network with over a million people using a smart thermometer to report temperature readings, about half also reported symptoms despite no clear incentive to do so. Consistent with heightened awareness of individual and public health needs, willingness to report symptoms was higher during the pandemic. And participants were willing to report even when they were not currently experiencing symptoms. Participants who were 50 to 65 years old or lived in a household with both adults and children were more likely to report their symptoms than those in other age groups or household configurations.

During the early stage of the COVID-19 pandemic, temperature-taking behavior was associated with both population disease levels and individual demographic characteristics^15^. Symptom-reporting behavior was found to be associated with individual demographic characteristics in flu seasons^13^. Prior studies found that women were more active in both online symptom reporting^12,22^ and health information seeking^23,24^. But even when females were more engaged in symptom reporting, they were less likely than males to have more than 2 additional follow-up symptom reports after the initial one^13^. Among our user base, females took more temperature readings and were more willing to report symptoms when taking temperature.

Consistent with prior research^4^, there was a higher likelihood of symptoms being reported when users first used the thermometer. Interestingly, symptom response rates dropped at the beginning of the pandemic and in the omicron BA.1 wave. This may relate to temperature-taking patterns. The number of episodes increased at the beginning of the pandemic, but the percentage of febrile episodes decreased, possibly driven by increased anxiety around infection, resulting in more temperature-taking. Early in the pandemic, higher coronavirus-related anxiety^25^ may explain the increased use of thermometers, and their use even when fevers were less likely to be present^7,18^.

This study has some limitations. First, self-reported symptoms without validation by physicians may contain inaccuracies. However, self-reported symptoms were reliable and valid in various clinical settings^26,27^, including early detection of ILI^28^ and COVID-19^29^. Self-reported symptoms have also been found valuable for infection surveillance^4,11,12,30^ and were both spatially and temporally correlated with the number of new of COVID-19 cases^8,31–35^.

Ability to participate in this network was subject to barriers that could potentially introduce bias. Smart thermometers are available in major retailers nationwide, but their higher cost compared to other options poses a financial burden. Reporting symptoms required a smartphone, excluding those without one. A digital divide among seniors, adults, and children could result in a higher likelihood of adults being participants in smartphone-based surveillance^12,31,36^. In our study, only 7% of participants were over 65 years old, and they were less likely to report symptoms after measuring their temperature. However, elderly individuals’ participation in surveillance has been trending upwards^37^. Children under 12 years old in our study were 35% of all participants, potentially over-represented as participants because of parental concerns. Young children and adults aged 50 and over had more episodes per person than other age groups, perhaps attributed to their health concerns.

To address potential bias and improve surveillance efforts targeting underserved populations, incorporating additional data collection methods using less advanced digital devices holds promise^9^. Undoubtedly, the presence of structural bias can introduce distortions in the results, particularly in regards to demographic, socioeconomic, and racial factors^38^ that can correlate with and contribute to disparities in health literacy^39^. However, targeted communication strategies and recruitment efforts can help mitigate these biases^14,40^. Further research is needed to gain a deeper understanding of specific factors that motivate and maintain engagement in participatory surveillance. Participants in the long-running Dutch Great Influenza Study (GIS)—which annually asks participants to report influenza-like symptoms to a central database and has a loyal and active group of participants^41^ —were motivated by being able to contribute to knowledge regarding flu, science, and the GIS project itself. This was especially true of more frequent reporters (2–3 times per month or weekly). Participants also reported learning something about flu (men more than women), although their factual knowledge was quite good. Similar surveys could help uncover background and motivating factors in the US, leading to greater representativeness in populations engaging in participatory surveillance.

For surveillance, other self-report approaches, such as large-scale surveys^8,42^, often have limitations related to participation and response. Digital data from search engines, social network systems, and smartphones, provided early indications of COVID-19^7,43,44^, but lacked information on participant characteristics, including demographics. Smart thermometers with a companion app provide a unique opportunity to combine automatically uploaded digital data with additional requested information, such as demographics and symptom reports, via the companion app. Future work could enhance the requested data to gain deeper insight regarding who participates and why.

We defined who belonged to a household based on use of a shared thermometer or smartphone for recording and uploading temperature readings. However, it is possible that other individuals within the same household used different thermometers, or did not participate in the surveillance network. This variability could introduce biases in the analyses concerning households.

Moreover, in certain situations that could potentially be severe, the app bypasses the symptom reporting page and redirects participants to a guidance page. While this could inadvertently lower the symptom reporting rate, participants retain the opportunity to document their symptoms at a later point. To streamline this process and ensure more comprehensive symptom data collection, we can integrate the symptom reporting step into the workflow when the guidance is needed. This could facilitate the capture of symptoms from these individuals.

The mitigating effect of vaccination on COVID-19 symptoms is well-documented^45,46^, underscoring a potential role of vaccination status in modifying symptom reporting behaviors^47,48^. It is conceivable that vaccinated individuals might perceive themselves as more protected, thereby diminishing their routine practices such as measuring their body temperature or utilizing relevant health tracking apps. Intriguingly, this anticipated behavior contrasted with our observation that individuals were more willing to report a symptom following the roll-out of vaccines during the winter wave, relative to earlier stages of the pandemic. To cultivate an enhanced understanding of how vaccination status influences symptom reporting, we propose implementing a follow-up question or disseminating a survey through the app. This could provide a more nuanced understanding of behavioral changes and their relation to the vaccination status of the users.

We were highly encouraged by the robust citizen participation in this network. They provided valuable insights for the designers regarding factors associated with engagement. The utilization of smart thermometers and accompanying apps for symptom collection provides a real-time, nationwide data source for disease monitoring, encompassing body temperatures, symptoms, demographic details, and geolocation information. It is essential to acknowledge that engagement with smart thermometer-based participatory syndromic surveillance systems may vary among people with diverse demographic characteristics and at different stages of a public health emergency. When utilizing data from these systems, it is crucial to account for these variations as they can have implications for data interpretation and analysis.

## Methods

### Design, setting, participants

This is a retrospective cohort study of a real-world, national-scale network of participants using commercial smart thermometers with a companion smartphone app that allows them to report demographics and symptoms (Kinsa Inc., San Francisco, California). Participants were individuals who used the thermometers and app between January 1, 2020 and October 29, 2022 and opted into data sharing. Participants identified in more than one household, defined as one or more individuals using the same thermometer or smartphone^21^, were excluded. This time span was divided into previously defined pandemic periods:^21,49–51^ before the COVID-19 pandemic in the United States (US) (January 1, 2020 to February 29, 2020); the first outbreak (March 1 to May 15, 2020); second period (May 16 to September 12, 2020); winter wave (September 13, 2020 to March 6, 2021); fourth period (March 7 to July 14, 2021); delta wave (July 15 to December 18, 2021); omicron BA.1/2 wave (December 19, 2021 to June 19, 2022); and omicron BA.4/5 wave (June 20, 2022 to October 29, 2022).

The number of newly activated smart thermometers–in other words, the number of new thermometers used for the first time–was included to analyze how popularity of smart thermometers affects the app survey response rate. For evaluating the relationship between new thermometers, number of temperature reading episodes, and cases of COVID-19, we obtained publicly available COVID-19 confirmed case counts in the United States from Johns Hopkins University, Center for Systems Science and Engineering (JHU, CSSE)^52^. The Boston Children’s Hospital IRB reviewed this study protocol and determined it to have exempt status because the study made secondary use of data which had been de-identified.

### Dependent, independent, and covariables

The main dependent variable is whether or not the presence of symptoms is reported, including reports of no symptoms, during an episode. Symptoms reportable via the app include cough, runny nose, body aches, headache, stuffy nose, chills, sore throat, fatigue, stomachache, diarrhea, vomiting, earache, loss of taste or smell, trouble breathing, nausea, rash, and seizure. As the pandemic progressed, loss of taste or smell was added as an option on June 9, 2020 and nausea added on August 9, 2021. The other symptoms were already present prior to the study period. Participants can also report that no symptoms are present. If participants do not report symptoms within an episode, the episode is coded as no response.

Independent variables are: season and pandemic period defined by the start date of the episode, febrile episode defined by temperature readings, whether an episode was from a new participant, self-reported age and gender, US Census divisions and Urban-Rural Classifications from automatically collected location data, and household composition.

Each participant’s temperature readings were grouped into episodes. If two readings were separated by more than 6 days, the later reading was treated as a separate episode. Fever was defined as a temperature of at least 38.0 °C for rectal and aural readings, 37.2 °C for axillary readings, and 37.8 °C for oral readings and other body sites^20,21^. Temperature readings outside the range of 34 °C to 43 °C were excluded. Fever onset in an episode was defined as the first body temperature at or exceeding the limits described above. A participant was considered new at the time of their first episode during the study period. Age and gender were self-reported. Geographic information was based on GPS information if participants opted into location sharing or an IP address associated with each temperature reading. Location was coded into nine geographic divisions based on US Census Bureau definitions^53^ and labeled with 2013 National Center for Health Statistics Urban-Rural Classifications^54^.

Episodes were excluded if multiple geographic divisions or urban-rural designations were coded or gender was missing in an episode. Household compositions were defined as having adults only, children only, or both children and adults. Households with children-only profiles were presumed to have an adult present who was not using the thermometer or app.

We captured information about when the symptom entry page was skipped due to severe fever, and incorporated it as a covariate. If a participant of any age has a high fever (at least 39.4 °C), or a participant aged 0–3 months has any fever, the symptoms report screen within the app is skipped and the user is immediately transferred to a guidance page to address potentially serious medical conditions. However, when the symptom reporting step is initially skipped, participants are still allowed to enter symptoms afterward.

### Statistical analysis

Baseline characteristics are presented as the median (IQR) for continuous variables and the number (%) for categorical variables. Differences in medians were assessed with the Kruskal-Wallis test because the variables did not follow a normal distribution. Chi-square test was used for univariate analysis of categorical variables. We performed mixed effects logistic regression to assess the factors affecting the engagement of symptom surveillance. Bonferroni correction was applied for multiple comparison testing. All analyses were performed using R (version 4.1.0, The R Foundation for Statistical Computing, www.r-project.org/). All statistical tests were two-sided, and statistical significance was defined as *p* < 0.05.

## Data Availability

Data will be made available to others upon request and upon completion of a data use agreement, only for research and non-commercial purposes, to individuals affiliated with academic or public health institutions.
